# RNA Captures More Cations than DNA: Insights from Molecular Dynamics Simulations

**DOI:** 10.1021/acs.jpcb.2c04488

**Published:** 2022-10-19

**Authors:** Sergio Cruz-León, Nadine Schwierz

**Affiliations:** † Department of Theoretical Biophysics, Max Planck Institute of Biophysics, Max-von-Laue-Str. 3, 60438Frankfurt am Main, Germany; ‡ Institute of Physics, University of Augsburg, Universitätsstraße 1, 86159Augsburg, Germany

## Abstract

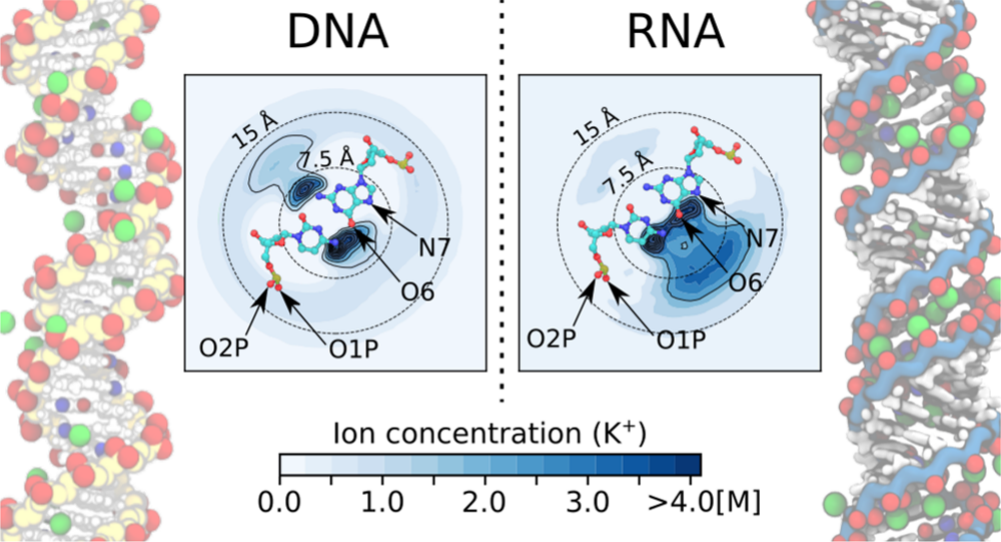

The distribution of cations around nucleic acids is essential for a broad variety of processes ranging from DNA condensation and RNA folding to the detection of biomolecules in biosensors. Predicting the exact distribution of ions remains challenging since the distribution and, hence, a broad variety of nucleic acid properties depend on the salt concentration, the valency of the ions, and the ion type. Despite the importance, a general theory to quantify ion-specific effects for highly charged biomolecules is still lacking. Moreover, recent experiments reveal that despite their similar building blocks, DNA and RNA duplexes can react differently to the same ionic conditions. The aim of our current work is to provide a comprehensive set of molecular dynamics simulations using more than 180 μs of simulation time. For the mono- and divalent cations Li^+^, Na^+^, K^+^, Cs^+^, Ca^2+^, Sr^2+^, and Ba^2+^, the simulations allow us to reveal the ion-specific distributions and binding patterns for DNA and RNA duplexes. The microscopic insights from the simulations display the origin of ion-specificity and shed light on the question of why DNA and RNA show opposing behavior in the same ionic conditions. Finally, the detailed binding patterns from the simulations reveal why RNA can capture more cations than DNA.

## Introduction

The distribution of cations around nucleic acids is essential for their structure and function. In electrolyte solutions, the highly charged backbone attracts an atmosphere of ions, which is responsible for electrostatic screening. In addition, a smaller fraction of cations binds specifically to ion binding sites.^1,2^ The most important ion binding sites are formed by the negatively charged phosphate oxygens and the nitrogens or oxygens on the nucleobases.^3−5^

The interdependence of cation-nucleic acid interactions, structure, and function is reflected in a wide variety of nucleic acid properties that depend on the electrostatic environment. One striking example is the salt dependence of DNA twist: DNA twist increases with increasing salt concentration and depends on the type of metal cation in solution. In particular, Ca^2+^ ions are the most efficient cations among alkali and alkaline earth cations to induce twist.^6−8^ Moreover, in biological systems, the ion atmosphere is essential to facilitate the folding of nucleic acids into functional three-dimensional structures.^9^ In these complex structures, the site-specific ions are typically coordinated by several nucleic acid atoms and play an important role in stabilization^5,9,10^ and facilitate chemical reactions, for instance in ribozymes.^11,12^

In addition to biological systems, the ion atmosphere is important in DNA-based technologies. For instance, biosensors that use an electric field to detect specific DNA sequences respond to, but also perturb, the ion atmosphere.^13,14^ Molecular insights into the distribution of ions around nucleic acids are therefore invaluable in understanding the role of cations in biological systems or optimizing the design of biosensors.

The distribution of cations and hence the electrostatic potential depends on the concentration of ions in solution, the valency of the ions, the type of ion, and the class of nucleic acid. While the former two can to some extent be captured by mean-field theories,^15,16^ ion-specific effects are more difficult to resolve. For nucleic acid systems, ion-specific effects are ubiquitous, and an increasing number of experimental results show that the stability, folding times, or reactivity depends not only on the concentration and valency of the ions but also on the ion type.^17−20^ However, to date, a general theory that quantifies ion-specific effects is still missing due to the complex interactions involved in ion binding and ion exchange.^21^

Moreover, the distributions of ions around DNA and RNA duplexes, which comprise the two most abundant structural motifs of nucleic acids, are not identical as might be expected from the similar compositions. As a consequence, DNA and RNA can show opposing behavior in the same ionic environment. For instance, and in contrast to DNA, RNA resists condensation,^22^ is stiffer in the presence of highly charged ions,^23,24^ and modifies its structure notably with ion type and concentration.^25,26^ One particularly striking result of recent ion-counting (IC) experiments, reflecting the different ion distributions around DNA and RNA, is that a simple RNA duplex consisting of only 24 base-pairs attracts more Na^+^ cations and expels fewer anions than the corresponding DNA duplex.^27^

A powerful tool to gain insights into the distribution of ions is all-atom molecular dynamics simulations in explicit water.^28^ These simulations allow us to characterize the interactions of cations and nucleic acids while including the subtle effects of ion hydration and water structure.^29^ However, simulating the distribution of ions remains challenging for two reasons. First, the simulations rely on accurate force fields for the nucleic acids, water, and ions. In particular, the force fields for the metal cations must be optimized to reproduce experimental solution properties^30,31^ and ion binding affinities^32−36^ in order to resolve the subtle differences between different cations. The second challenge for simulations is that for cation binding, the transition from a water-mediated outer-sphere to a direct inner-sphere coordination is on the micro- to millisecond time scale for metal cations with high charge density such as Mg^2+^.^11,37−39^ Therefore, simulating an equilibrated distribution for highly charged ions is out of reach for conventional simulation techniques, and enhanced sampling schemes need to be applied.^34,38,40^ Here, the quantitative comparison of experiments, simulations, and theoretical modeling is essential to drive the continuous improvement of atomistic models and theoretical methods.^8,41−44^ In turn, simulations can contribute significantly to a deeper understanding of the interactions between cations and nucleic acids and reveal the selectivity of cation binding sites,^17,45^ the sequence dependence of ion binding affinities,^46^ the influence of the handedness,^47^ or ion competition.^42−44^

Since the work by Bai et al.^41^ demonstrating the existence of ion-specific effects in the ionic atmosphere around DNA, multiple experimental^8,25−27,48,49^ and computational^2,8,42−44,46,47,50^ efforts aimed at understanding cation-nucleic acid interactions. For instance, IC experiments were used to test the accuracy of MD simulations.^42−44^ However, most of the MD studies were restricted to short length and time scales, which limited their predictability on the binding patterns and nucleic acid structures. The cations arrange according to the nucleic acid structure,^2,51^ and simultaneously, the structure of the nucleic acid changes with the ionic environment.^8,25,26^ Recently, it has been reported that several helical turns are required to quantify structural changes of the nucleic acids^52,53^ and that hundreds of ns were required to obtain converged K^+^ distributions.^46^ Moreover, cations with high charge density and slow exchange kinetics will exacerbate this convergence limitation further.^17^ Resolving ion-specific nucleic acid interactions thus requires multi-μs simulations on multiple turn helices.

The aim of this work is to resolve the origin of ion-specific and nucleic-acid-specific distributions of cations around DNA and RNA duplexes. We complement the existing efforts from the literature^42−47,50,54−56^ by providing a comprehensive and extensive set of MD simulations using more than 180 μs of simulation time. We provide detailed insights into the ion-specific distribution and binding patterns for a large group of mono- and divalent metal cations (Li^+^, Na^+^, K^+^, Cs^+^, Ca^2+^, Sr^2+^, and Ba^2+^). Finally, the molecular insights allow us to resolve the question of why and for which type of cations RNA can capture more cations than DNA.

## Methods

### Atomistic Simulations

We performed unrestrained simulations of a 33-base-pair (bp) DNA or RNA duplex in B-helix and A-helix forms, respectively. For DNA, we analyzed the MD simulations reported in our earlier work,^8^ performed with a similar methodology as the one use here for RNA and described below. For RNA, we performed additional simulations using the corresponding RNA sequence 3′-GAGAU-GCUAA-CCCUG-AUCGC-UGAUU-CCUUG-GAC-5′, in its canonical A-helix form. The structure of RNA was generated using the nucleic acid builder software.^57^

Briefly, the RNA duplexes were simulated with LiCl, NaCl, KCl, CsCl, CaCl_2_, SrCl_2_, and BaCl_2_ at concentrations of 100, 250, 500, and 1000 mM for monovalent cations and 25, 50, and 100 mM for divalent cations. The duplex was placed in an orthorhombic dodecahedron box assuring a minimal distance of 2 nm to the edge and filled with TIP3P water molecules.^58^ A typical simulation system is shown in Figure S1. After the pre-equilibration, production runs were performed with a time step of 2 fs in the NPT ensemble using the isotropic Parrinello Rahman barostat^59^ with a coupling constant of 5.0 ps and the velocity rescaling thermostat with a stochastic term.^60^ The simulations for mono- and divalent cations were 3 μs and 5 μs long, respectively. The RNA was described with the Amber force field parmbsc0 + χ_0*L*3_.^61−63^ Our previous simulations^8^ described DNA with the Amber force field parmbsc1.^64^

For both cases, DNA and RNA, the metal cations were described using the force field developed by Mamatkulov and Schwierz^30^ and its subsequent extension for Ca^2+^ interacting with nucleic acids.^33^

We selected a large variety of alkali and alkaline earth cations, which are frequently used for *in vitro* assays in biotechnology and allow us to resolve ion-specific effects. The choice of ion force field was motivated by the fact that the optimized parameters yield accurate ion-pairing properties as judged by comparison to experimental activity coefficients and accurate exchange kinetics as judged by experimental water exchange rates.^30^ In particular, the parameters were shown to resolve the fine differences between distinct metal cations.^8,17,29^ Note that Mg^2+^ was excluded from the present study. For Mg^2+^, the transition from the water-mediated outer-sphere to the inner-sphere coordination is on the micro- to millisecond time scale.^38,39^ It is, therefore, tremendously challenging to obtain an equilibrated distribution for Mg^2+^ with the available computational resources.

For the analysis, the first 200 ns was discarded for equilibration, and the last three bases at each end were not considered. Further details on the simulation protocol can be found in the Supporting Information and in ref (8).

### Poisson–Boltzmann Modeling

We complemented our simulations with Poisson–Boltzmann (PB) theory. In particular, we used an extended PB equation,^65,66^ which includes the ion-nucleic acid interaction potentials (PMFs) derived from MD simulations.^17^ This approach has been successfully applied in our previous work to predict the results from ion-counting experiments and the competition between ions in the ionic atmosphere around DNA.^17^ Here, the duplexes were modeled as cylinders with radius *R*_RNA_ = 1.3 or *R*_DNA_ = 1.0 nm, corresponding to the A-helix and B-helix forms. We used a constant surface charge density of σ_RNA_= −0.83 *e*/nm^2^ or σ_DNA_= −0.89 *e*/nm^2^. The PB equation was solved numerically, as described in ref (17), yielding the concentration profiles *c*_*i*_(*r*) as a function of the distance *r*. We solved the PB equation for different ions at different bulk salt concentrations and obtained the corresponding ion excess as described below.

### Ion Distributions and Excess

We also calculated the ion concentration profiles from the MD trajectories using untwisted curvilinear helicoidal coordinates. The coordinate system, implemented in the module canion of the software Curves+,^46,50^ has the same symmetry as the nucleic acid duplexes. With this method, one can obtain a detailed characterization of the local interactions, smeared out when conventional Cartesian averages are used.^50^ We provide two complementary representations: one-dimensional concentration profiles *c*_*i*_(*r*) and two-dimensional untwisted distributions projected on the *x*–*y* plane. See details in the Supporting Information.

From the ion concentrations *c*_*i*_(*r*), we obtained the excess/depletion of ions Γ_*i*_ assuming an infinite cylinder:
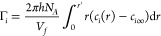
1where *N*_*A*_ is Avogadro’s number, *V*_*f*_ is a conversion factor for consistent units, *c*_*i∞*_ is the bulk ion concentration obtained at large *r*, and *h* is end-to-end length of the helix. Note that we chose a large enough simulation box (Figure S1) such that (*c*_*i*_(*r*) – *c*_*i∞*_) goes to zero for large distances (Figures S2 and S3).

The systems fulfill the electroneutrality condition
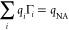
2where *q*_*i*_ is the charge of the ion species *i*, and *q*_NA_ = 64 is the absolute value of the total charge of the nucleic acid.

As in the experimental IC data, we calculate the fraction Γ_i_^*^ of attracted cations and excluded anions per negative charge on the phosphate groups of the nucleic acid molecules
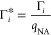
3

### Ion Binding Patterns

We identified the ion binding sites and the emerging binding patterns. To that end, we followed individual time series of the distances (*d*_*mj*_) between each of the *N*_*m*_ cations in the simulation box and all oxygen and nitrogens atoms *j* of the DNA or RNA. Note that one cation can be coordinated by several nucleic acid atoms simultaneously. From the distances, we determined the set of nucleic acid atoms *x* = {*j*, *k*, ...} that are within a cutoff distance from the cations *m*.

The values for the cutoff *d*^†^ are shown in Table S1 and are based on our previous work.^17^ Hereby, the cutoff was chosen such that only inner-sphere binding (i.e., a direct contact between the ion and the atoms of the nucleic acid) was taken into account. Outer-sphere interactions are not included in our current analysis. Our choice is motivated by the fact that they are only transient and exchange fast with the surrounding solvent.^17^ In addition, the binding affinity of inner-sphere binding is significantly higher compared to outer-sphere binding such that inner-sphere binding dominates in most cases^17,33^ (see also Figures S4 and S5). However, note that outer-sphere interaction can play an important role, for example, in stabilizing tertiary contacts in RNA as revealed by X-ray structures.^67^ Finally, the probability *p*_*x*_ of a binding pattern was calculated and corresponds to the probability of an ion to be coordinated by nucleic acid atoms *x* = {*j*, *k*, ...}. *p*_*x*_ was obtained from the frequency of pattern *x* and normalized by the total number of cations *N*_*m*_ and number of simulation frames.

## Results and Discussion

This work aims to gain insights into ion-specific cation-DNA and cation-RNA interactions by resolving the ion distributions and ion binding patterns. The molecular insights gained from the simulations reveal why RNA—despite having the same charge—can capture more monovalent cations than DNA.

### RNA Captures More Na^+^ than DNA

**Figure 1:**
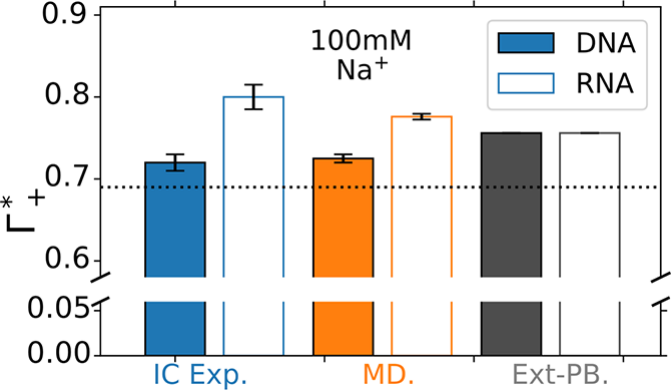
Comparison of simulations and experiments. Fraction of attracted Na^+^ ions Γ_+_^*^ for DNA (open filled) and RNA (open bars) in 100 mM NaCl from ion-counting experiments,^27^ MD simulations, and extended PB theory. Errors in the simulations were obtained from block averaging. The dotted horizontal line is the result from standard PB theory.

Recent ion-counting experiments show that an RNA duplex attracts more cations and expels fewer anions compared to a DNA duplex.^27^Figure 1 compares the results from these experiments, namely the fraction of attracted cations Γ_+_^*^, with the results from PB theory and MD simulations. The results show that PB theory does not capture the differences between DNA and RNA. This result illustrates that the structural differences between DNA and RNA helices, which are not included in our approach for Poisson–Boltzmann theory, have to be included to predict the experimentally observed differences.

On the other hand, the MD simulations are particularly well suited and correctly reproduce the ion-counting experiments (Figure 1). Given the remarkable agreement at 100 mM, the simulations are ideal for providing further microscopic insights into the question of why the RNA duplex attracts more cations than the DNA duplex.

**Figure 2:**
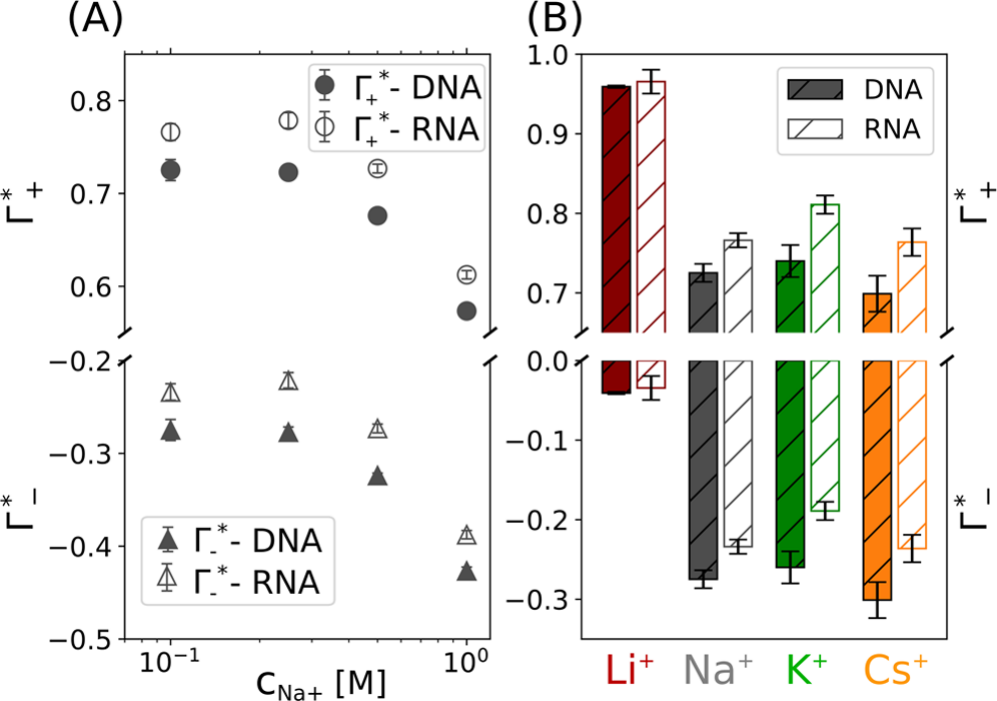
Ion excess of DNA and RNA. (A) Fraction of attracted and repelled ions Γ_±_^*^ as a function of NaCl concentration for DNA (filled symbols) and RNA (open symbols) obtained from MD simulations. (B) Γ_±_^*^ obtained at 100 mM monovalent salt concentration for LiCl, NaCl, KCl, and CsCl with DNA (filled hatched bars) or RNA (open hatched bars). Error bars were obtained from block averaging.

Figure 2A shows the fraction of attracted cations  and depleted anions  from the simulations as a function of the NaCl salt concentration. As expected, the excess of cations decreases as the bulk concentration increases. In agreement with the ion-counting measurements,^27^ the RNA captures more Na^+^ cations and depletes fewer Cl^–^ anions than DNA over the full concentration range .

Figure 2B gives insights into ion-specific effects: For K^+^ and Cs^+^, the fraction of attracted cations and depleted anions is similar to Na^+^ (Figure 2B). In all three cases, RNA captures more cations than DNA . Overall, these results agree with the ion competition measurements, which do not reveal a large difference between those ions^41,49^ and coincide with the most recent measurements for Na^+^ and Cs^+^.^27^ By contrast, Li^+^ plays a distinct role. The excess of Li^+^ in our simulations is about 20% higher compared to the other monovalent ions, in qualitative agreement with experiments with DNA where  is ∼10% higher than .^49^ Furthermore, the differences between DNA and RNA diminish. The distinct role of Li^+^ has also been observed in ion-counting experiments,^41,49^ nanopore translocation experiments,^68^ or DNA twist.^8^

Continuous validation of the force fields for metal cations is essential to improve the agreement between simulations and experiments. Unfortunately, experimental data sets in particular for divalent ions are often scarce.^41,69,70^ Moreover, ion competition measurements, as in the work by Bai et al.,^41^ provide a different condition that cannot be directly compared to our MD simulations.^17^ In addition, low ion concentrations are challenging in the simulations, since they require large systems sizes and long simulation times to obtain equilibrated distributions. Therefore, the comparison of the divalent ions will require additional experimental data to establish a feedback loop for validation.

### Ion-Specific and Nucleic-Acid-Specific Distribution of Cations

**Figure 3:**
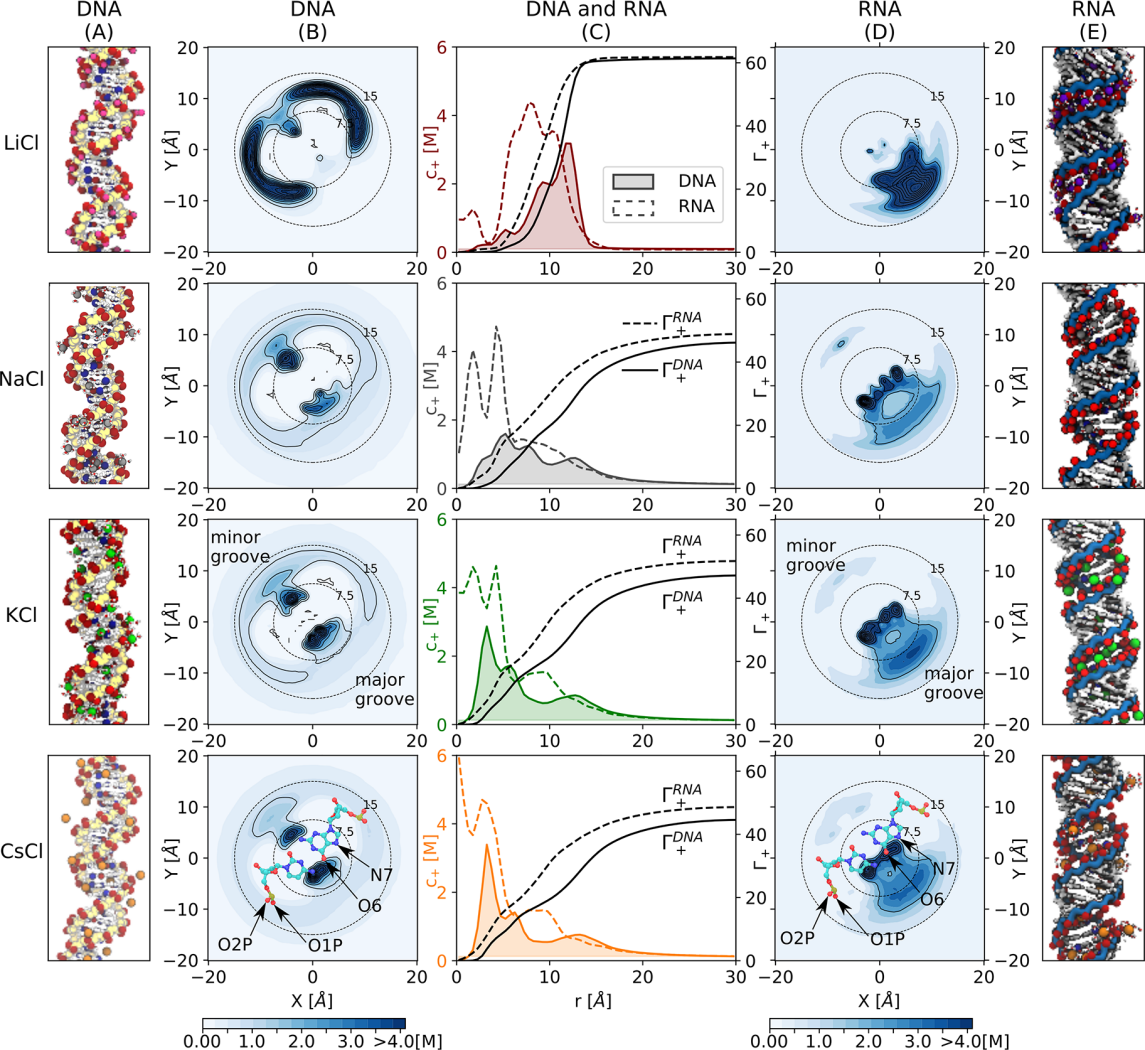
Ion distribution and excess of monovalent cations around DNA and RNA. (A,E) Simulation snapshots. Backbone atoms are indicated in yellow and blue for DNA and RNA, respectively. The most frequent ion binding sites are highlighted: red (O1P, O2P, and O6 atoms) and blue (N7 atoms). (B,D) Top view of the untwisted helicoidal ion concentration obtained with the software canion.^46,50^ In this representation, the upper-left and lower-right corners correspond to the minor and major grooves as indicated by the superimposed molecular schemes of cytosine-guanine (bottom). In these schemes, the most frequent ion binding sites are labeled. The dotted concentric circles indicate the distance to the center of the helix (radius in Å). (C) Ion concentration profiles *c*_+_ as a function of the distance *r* and cation excess Γ_+_(*r*) obtained from eq 1 for DNA (solid line) and RNA (dashed line). Concentration profiles for DNA are filled for clarity.

**Figure 4:**
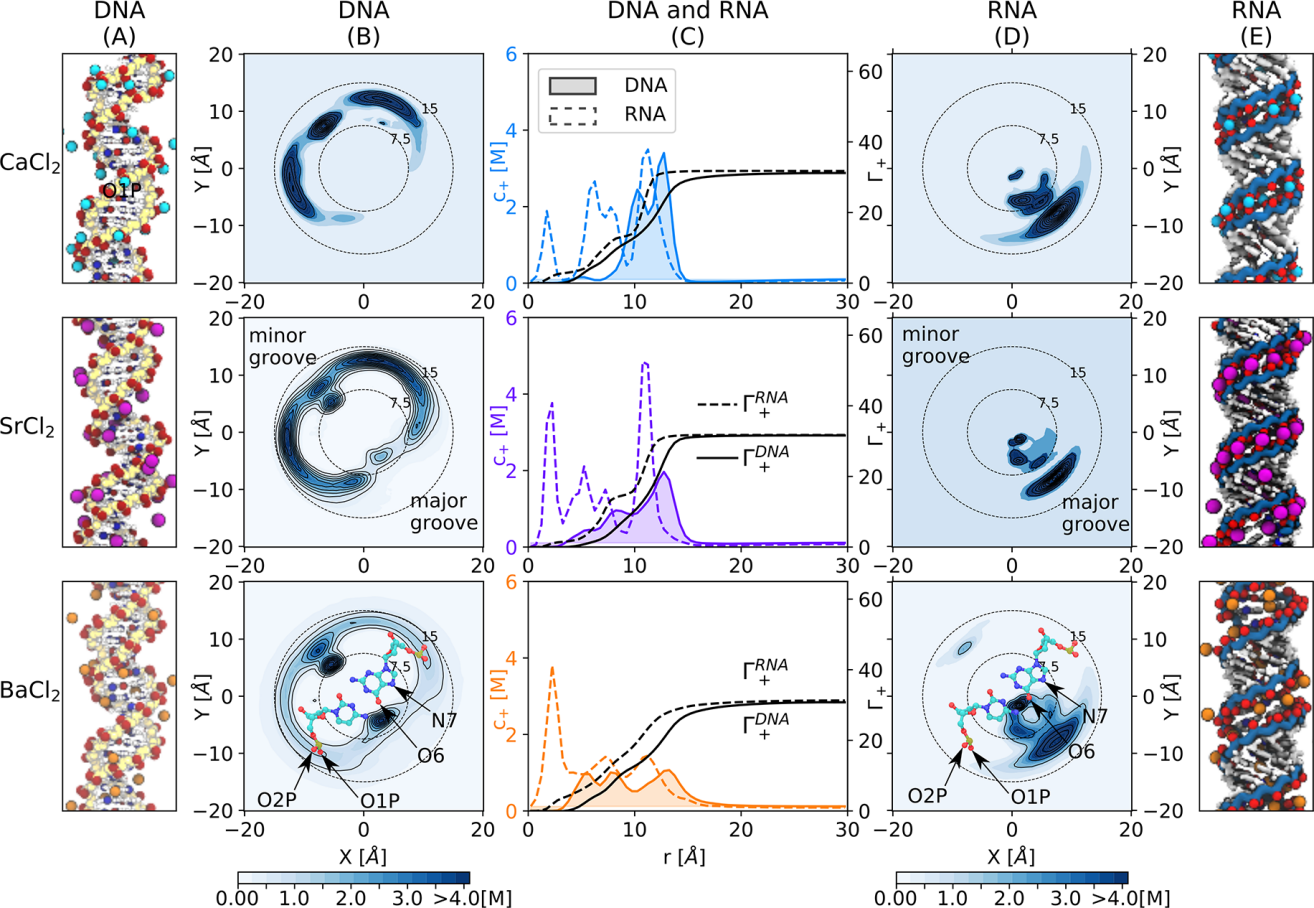
Ion distribution and excess of divalent cations around DNA and RNA. (A,E) Simulation snapshots. Backbone atoms are indicated in yellow and blue for DNA and RNA, respectively. The most frequent ion binding sites are highlighted: red (O1P, O2P, and O6 atoms) and blue (N7 atoms). (B,D) Top view of the untwisted helicoidal ion concentration obtained with the software canion.^46,50^ In this representation, the upper-left and lower-right corners correspond to the minor and major grooves as indicated by the superimposed molecular schemes of cytosine-guanine (bottom). In these schemes, the most frequent ion binding sites are labeled. The dotted concentric circles indicate the distance to the center of the helix (radius in Å). (C) Ion concentration profiles *c*_+_ as a function of the distance *r* and cation excess Γ_+_(*r*) obtained from eq 1 for DNA (solid line) and RNA (dashed line). Concentration profiles for DNA are filled for clarity.

Figures 3 and 4 show the distributions of the mono- and divalent ions around DNA and RNA.

In general, the ionic atmosphere consists of site-specific and diffusive ions. The latter leads to a monotonic decay/increase of the cation/anion concentration toward the bulk (Figures 3C and 4C) as predicted by classical mean-field theories,^15,16^ and it is similar at all studied concentrations (Figures S2 and S3). In the vicinity of the nucleic acids, the specific interactions between the cations and the nucleic acids lead to unique patterns. These binding patterns clearly depend on the ion type and are different for DNA and RNA (Figures 3B–D and 4B–D).

For example, the distributions of cations around DNA have a local density maximum at the position of the phosphate oxygens of the backbone, indicating preferential interactions of the cations and the phosphate oxygens (Figures 3B,C, 4B,C, S4, and S5). The intensity of the peak and therefore the accumulation of the cations is higher for ions with high charge density (Li^+^ or Ca^2+^) compared to ions with low charge density (K^+^ or Cs^+^).

By contrast, the innermost peak (at *r* < 10 Å in Figures 3B and 4B), which corresponds to the interaction with the nucleobases at the minor and major grooves, shows the opposite trend: Here the density increases with decreasing ion charge density (Li^+^ < Na^+^ < K^+^ < Cs^+^and Ca^2+^ < Sr^2+^ < Ba^2+^). These trends are identical for all concentrations (Figures S1 and S2) and reflect the binding affinity of the ions to the backbone and nucleobase binding sites:^17^ Ions with high charge density (such as Li^+^ or Ca^2+^) have a higher binding affinity to the phosphate oxygens, while cations with low charge density (such as Cs^+^) have a higher binding affinity toward the N7 and O6 of the nucleobases (see also S4 and S5).

Despite the identical charge and sequence of DNA and RNA, the cation distribution and binding patterns are remarkably different (Figures 3 and 4). The differences are caused by the different topologies: DNA forms a B-helix, while RNA forms an A-helix. For DNA, the cations are localized in the major groove, in the minor groove, and at the backbone, since the preferential ion binding sites (in particular phosphate oxygen, O6, and N7 atoms) are segregated due to the B-form (Figures 3A and 4A).

For RNA, in addition to the nucleobase binding sites (N7 and O6 atoms) that are located in the major groove, the phosphate oxygens of the backbone point toward the interior of the major groove (Figures 3E and 4E). The close proximity of the partially charged atoms creates a high electrostatic potential that attracts and traps the cations inside of the major groove. This observation is in agreement with the so-called electrostatic focusing found in previous work.^71^

In summary, the different nucleic acid topologies give rise to unique cation distributions around DNA and RNA. For DNA, the preferential ion binding sites are segregated, and the ions are distributed in the major groove, the minor groove, and around the backbone according to their binding affinities at the individual sites. For RNA, the ion binding sites are in close proximity, leading to a high local electrostatic potential and an accumulation of the cations in the major groove.

### Ion Binding Patterns of DNA and RNA

**Figure 5:**
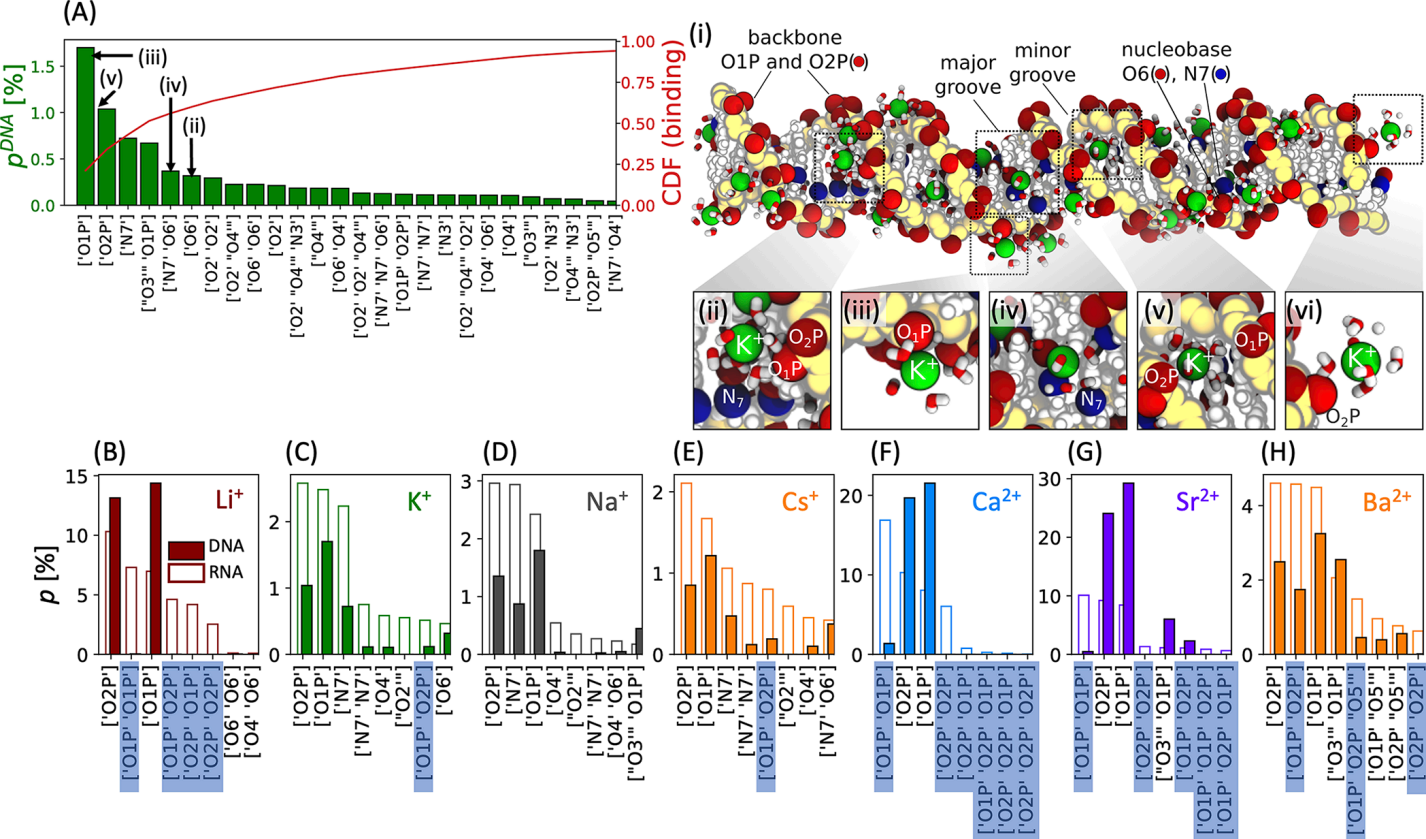
Ion binding patterns for DNA and RNA. (A) Probability *p*^DNA^ and cumulative distribution function of a given binding pattern for DNA in 100 mM KCl. The binding patterns on the *x*-axis indicate the nucleic acid atoms that form an inner-sphere contact with the cation. (i–vi) Simulation snapshots of K^+^-DNA interactions. Panel (vi) shows an example of outer-sphere binding. Note that outer-sphere binding is not included in our analysis. Backbone atoms are indicated in yellow, and the most frequent ion binding sites are highlighted: red (O1P, O2P, and O6 atoms) and blue (N7 atoms). K^+^ (green) and its first hydration shell are shown. (B–H) Probability *p* of a given binding pattern for DNA (solid bars) and RNA (open bars). Only the highest eight binding probabilities for RNA are shown and compared to DNA. The binding patterns marked with a blue box indicate intramolecular binding (i.e., bridging between the two strands of the same DNA or RNA molecule).

Figure 5 shows the ion binding patterns for DNA and RNA. The binding patterns are defined by the atoms of the nucleic acids that are in direct contact with the cations (inner-sphere coordination). Hereby, the cations can be coordinated transiently by up to five nucleic acid atoms. The most frequently occurring atom types are the phosphate oxygens (O1P and O2P) and, for the nucleobases, the N7 and O6 atoms on guanine, O4 on thymine and uracil, and O2 on cytosine. The oxygen atoms O3′, O4′, and O5′ of the sugar also appear but less frequently. Note that these atom types are identical to the most important ion binding sites on RNA that were previously identified experimentally.^3,4^

The binding patterns (Figure 5B–H) are ion-specific and diverse. Still, a few repeating patterns explain almost all binding events. For instance, out of the 26 binding patterns for K^+^ at DNA, the first 6–8 binding patterns account for about 70% of binding events as shown by the cumulative distribution function (Figure 5A).

Comparing the binding patterns of DNA and RNA reveals similarities and differences (Figures 5B–H and S6). In both cases, the cations preferentially interact with the phosphate oxygens (O1P and O2P) of the backbone. In addition, cations with low charge density interact with the N7, O6, and O4 atoms as expected from the ion distributions. Interestingly, it is not evident that the lack of the 2′-OH group on DNA, which is one of the crucial differences from RNA, plays a direct role in the different ion binding patterns (Figures 5B–H and S6). However, the 2′-OH group leads to different structures of the sugar moiety and to the A- and B-helix structures of RNA and DNA. It therefore modifies the nucleic acid structures and hence indirectly the ion binding patterns.

The most striking difference is that the cations are coordinated mainly by a single nucleic acid atom for DNA, while for RNA, coordination by multiple atoms occurs often. For example, the cations can form intramolecular zippers resulting in a simultaneous coordination by two phosphate oxygens. This bridging results in the closing of the major groove and is predominantly observed for cations with high charge density such as Li^+^, Sr^2+^, and Ca^2+^ (see also snapshots in Figure 4E). In addition, cations with low charge density Na^+^, K^+^, and Cs^+^ can be simultaneously coordinated by a phosphate oxygen and nucleobase atoms, in particular O6 or N7. The binding affinity of such multicoordinated configurations is considerably higher compared to the situation where only one coordinating atom is involved. The formation of multicoordinated configurations therefore explains why an RNA duplex captures more (low charge density) cations compared to DNA. However, for cations with high charge density, direct inner-sphere interactions with the nucleobase atoms are unfavorable^17,45^ (see also S4 and S5). Instead, those cations interact strongly with the O1P or O2P and saturate the ionic atmosphere, and the differences in the numbers of captured cations for DNA and RNA diminishes.

The ion-specific distributions and ion binding patterns around DNA and RNA help to understand a broad range of experiments in which nucleic acids respond differently to changes in the ionic environment. One example is DNA condensation in the presence of highly charged cations.^72^ The effective attraction emerges from the cation-mediated interhelix bridging of phosphate groups.^73,74^ By contrast, RNA resists condensation.^22^ Our results show that for RNA the cations are located within the major groove (Figure 3D) and form an intramolecular zipper-like closing of the major groove (Figure 3E). The resulting inward location of the cations hinders intermolecular cation-bridging such that RNA resists condensation. Another example is the opposing effects of multivalent ions on the flexibility of DNA and RNA.^23,24^ Here, we note that for high-affinity ions, our results show the intramolecular zipper-like closing of the major groove. This effect is similar to the effect recently reported by Fu et al.^24^ for multivalent ions. In their case, this leads to the stiffening of the helix and the observed loss in elasticity.

## Conclusions

Predicting the exact distribution of ions around DNA and RNA remains challenging, since the distribution depends on the salt concentration, the valency of the ions, the ion type, and the type of nucleic acid. The aim of our current work is to provide molecular insights into the ion-specific distributions of seven different metal cations around DNA and RNA duplexes. Our results show that molecular dynamics simulations are a powerful tool to gain insights. Moreover, using optimized force fields for the cations^30,33^ allows us to resolve the subtle differences between the ions and to yield close agreement with the results from ion-counting experiments.^27^ The simulations reveal that the ion distributions and binding patterns for DNA and RNA are remarkably different. For DNA, the ion binding sites are segregated, and the ions are distributed in the major groove, the minor groove, and around the backbone according to the binding affinities at the individual sites composed of phosphate oxygens or N7, O6, and O4 atoms on the nucleobases. For RNA, the ion binding sites are in close proximity, leading to a high local electrostatic potential and an accumulation of the cations in the major groove. These distinct distributions result in strikingly different and ion-specific binding patterns. For DNA, the cations are typically coordinated by one nucleic acid atom. For RNA, the ions are coordinated by multiple nucleic acid atoms leading to a zipper-like closing of the major groove for cations with high charge density such as Li^+^, Ca^2+^, and Sr^2+^ or an accumulation of cations such as Na^+^, K^+^, and Cs^+^ inside the major groove. This formation of high affinity multicoordinated configurations inside the major groove, which is not possible for DNA, explains the differences in the number of captured ions for the two types of nucleic acids.

In summary, MD simulations allow us to reveal the microscopic origin of ion-specificity and shed light on the question of why DNA and RNA show opposing behavior in the same electrostatic environment. However, to generalize these findings and to predict ion-specific distributions around arbitrarily charged biomolecules, more work and reliable experimental data are required. In this respect, the data provided here may serve as a valuable starting point to assess and validate theoretical models. Finally, our results show that ion-specific effects change the electrostatics around nucleic acids. The resulting differences could be used for the design of of ion-specific trapping devices.

## Supporting Information Available

Additional simulation details. Concentration profiles for all the ions at different bulk concentrations. Radial distribution functions between all cations and the main binding sites of the backbone and nucleobases of DNA and RNA. Probability of the ion binding patterns of DNA and RNA (extended) (PDF)

## References

[ref1] Draper D. E. A guide to ions and RNA structure. RNA 2004, 10, 335–343. 10.1261/rna.5205404. 14970378 PMC1370927

[ref2] Lipfert J.; Doniach S.; Das R.; Herschlag D. Understanding Nucleic Acid–Ion Interactions. Annu. Rev. Biochem. 2014, 83, 813–841. 10.1146/annurev-biochem-060409-092720. 24606136 PMC4384882

[ref3] Sigel R. K.; Sigel H. A stability concept for metal ion coordination to single-stranded nucleic acids and affinities of individual sites. Acc. Chem. Res. 2010, 43, 974–984. 10.1021/ar900197y. 20235593

[ref4] Auffinger P.; Grover N.; Westhof E. Metal ion binding to RNA. Met Ions Life Sci. 2011, 9, 1–35. 10.1039/9781849732512-00001. 22010267

[ref5] Sigel R. K.; Pyle A. M. Alternative roles for metal ions in enzyme catalysis and the implications for ribozyme chemistry. Chem. Rev. 2007, 107, 97–113. 10.1021/cr0502605. 17212472

[ref6] Anderson P.; Bauer W. Supercoiling in closed circular DNA: dependence upon ion type and concentration. Biochemistry 1978, 17, 594–601. 10.1021/bi00597a006. 623732

[ref7] Xu Y.-C.; Bremer H. Winding of the DNA helix by divalent metal ions. Nucleic Acids Res. 1997, 25, 4067–4071. 10.1093/nar/25.20.4067. 9321659 PMC147022

[ref8] Cruz-León S.; Vanderlinden W.; Müller P.; Forster T.; Staudt G.; Lin Y.-Y.; Lipfert J.; Schwierz N. Twisting DNA by salt. Nucleic Acids Res. 2022, 50, 5726–5738. 10.1093/nar/gkac445. 35640616 PMC9177979

[ref9] Woodson S. A. Metal ions and RNA folding: a highly charged topic with a dynamic future. Curr. Opin. Chem. Biol. 2005, 9, 104–109. 10.1016/j.cbpa.2005.02.004. 15811793

[ref10] Pyle A. Metal ions in the structure and function of RNA. J. Biol. Inorg. Chem. 2002, 7, 679–690. 10.1007/s00775-002-0387-6. 12203005

[ref11] Freisinger E.; Sigel R. K. From nucleotides to ribozymes—a comparison of their metal ion binding properties. Coord. Chem. Rev. 2007, 251, 1834–1851. 10.1016/j.ccr.2007.03.008.

[ref12] Schnabl J.; Sigel R. K. Controlling ribozyme activity by metal ions. Curr. Opin. Chem. Biol. 2010, 14, 269–275. 10.1016/j.cbpa.2009.11.024. 20047851

[ref13] Stern E.; Wagner R.; Sigworth F. J.; Breaker R.; Fahmy T. M.; Reed M. A. Importance of the Debye screening length on nanowire field effect transistor sensors. Nano Lett. 2007, 7, 3405–3409. 10.1021/nl071792z. 17914853 PMC2713684

[ref14] Bronder T. S.; Poghossian A.; Scheja S.; Wu C.; Keusgen M.; Mewes D.; Schöning M. J. DNA immobilization and hybridization detection by the intrinsic molecular charge using capacitive field-effect sensors modified with a charged weak polyelectrolyte layer. ACS Appl. Mater. Interfaces 2015, 7, 20068–20075. 10.1021/acsami.5b05146. 26327272

[ref15] Manning G. S. Limiting laws and counterion condensation in polyelectrolyte solutions I. Colligative properties. J. Chem. Phys. 1969, 51, 924–933. 10.1063/1.1672157.

[ref16] Deserno M.; Holm C.; May S. Fraction of Condensed Counterions around a Charged Rod: Comparison of Poisson-Boltzmann Theory and Computer Simulations. Macromolecules 2000, 33, 199–206. 10.1021/ma990897o.

[ref17] Cruz-León S.; Schwierz N. Hofmeister Series for Metal-Cation–RNA Interactions: The Interplay of Binding Affinity and Exchange Kinetics. Langmuir 2020, 36, 5979–5989. 10.1021/acs.langmuir.0c00851. 32366101 PMC7304902

[ref18] Vieregg J.; Cheng W.; Bustamante C.; Tinoco I. Measurement of the Effect of Monovalent Cations on RNA Hairpin Stability. J. Am. Chem. Soc. 2007, 129, 14966–14973. 10.1021/ja074809o. 17997555 PMC2528546

[ref19] Koculi E.; Hyeon C.; Thirumalai D.; Woodson S. A. Charge Density of Divalent Metal Cations Determines RNA Stability. J. Am. Chem. Soc. 2007, 129, 2676–2682. 10.1021/ja068027r. 17295487 PMC2523262

[ref20] Fang X.-W.; Thiyagarajan P.; Sosnick T. R.; Pan T. The rate-limiting step in the folding of a large ribozyme without kinetic traps. Proc. Natl. Acad. Sci. U. S. A. 2002, 99, 8518–8523. 10.1073/pnas.142288399. 12084911 PMC124294

[ref21] Schwierz N.; Horinek D.; Sivan U.; Netz R. R. Reversed Hofmeister series—The rule rather than the exception. Curr. Opin. Colloid Interface Sci. 2016, 23, 10–18. 10.1016/j.cocis.2016.04.003.

[ref22] Li L.; Pabit S. A.; Meisburger S. P.; Pollack L. Double-stranded RNA resists condensation. Phys. Rev. Lett. 2011, 106, 108101 10.1103/PhysRevLett.106.108101. 21469837 PMC3156472

[ref23] Drozdetski A. V.; Tolokh I. S.; Pollack L.; Baker N.; Onufriev A. V. Opposing Effects of Multivalent Ions on the Flexibility of DNA and RNA. Phys. Rev. Lett. 2016, 117, 028101 10.1103/PhysRevLett.117.028101. 27447528 PMC5493319

[ref24] Fu H.; Zhang C.; Qiang X.-W.; Yang Y.-J.; Dai L.; Tan Z.-J.; Zhang X.-H. Opposite Effects of High-Valent Cations on the Elasticities of DNA and RNA Duplexes Revealed by Magnetic Tweezers. Phys. Rev. Lett. 2020, 124, 058101 10.1103/PhysRevLett.124.058101. 32083903

[ref25] Chen Y.-L.; Pollack L. Salt dependence of A-form RNA duplexes: structures and implications. J. Phys. Chem. B 2019, 123, 9773–9785. 10.1021/acs.jpcb.9b07502. 31638810 PMC7068736

[ref26] He W.; Chen Y.-L.; Pollack L.; Kirmizialtin S. The structural plasticity of nucleic acid duplexes revealed by WAXS and MD. Sci. Adv. 2021, 7, eabf6106 10.1126/sciadv.abf6106. 33893104 PMC8064643

[ref27] Gebala M.; Herschlag D. Quantitative Studies of an RNA Duplex Electrostatics by Ion Counting. Biophys. J. 2019, 117, 1116–1124. 10.1016/j.bpj.2019.08.007. 31466697 PMC6818163

[ref28] Šponer J.; Bussi G.; Krepl M.; Banáš P.; Bottaro S.; Cunha R. A.; Gil-Ley A.; Pinamonti G.; Poblete S.; et al. RNA Structural Dynamics As Captured by Molecular Simulations: A Comprehensive Overview. Chem. Rev. 2018, 118, 4177–4338. 10.1021/acs.chemrev.7b00427. 29297679 PMC5920944

[ref29] Van Lin S. R.; Grotz K. K.; Siretanu I.; Schwierz N.; Mugele F. Ion-specific and ph-dependent hydration of mica–electrolyte interfaces. Langmuir 2019, 35, 5737–5745. 10.1021/acs.langmuir.9b00520. 30974056 PMC6495383

[ref30] Mamatkulov S.; Schwierz N. Force fields for monovalent and divalent metal cations in TIP3P water based on thermodynamic and kinetic properties. J. Chem. Phys. 2018, 148, 074504 10.1063/1.5017694. 29471634

[ref31] Fyta M.; Kalcher I.; Dzubiella J.; Vrbka L.; Netz R. R. Ionic force field optimization based on single-ion and ion-pair solvation properties. J. Chem. Phys. 2010, 132, 024911 10.1063/1.3292575. 20095713

[ref32] Panteva M. T.; Giambaşu G. M.; York D. M. Force field for Mg^2+^, Mn^2+^, Zn^2+^, and Cd^2+^ ions that have balanced interactions with nucleic acids. J. Phys. Chem. B 2015, 119, 15460–15470. 10.1021/acs.jpcb.5b10423. 26583536 PMC4762653

[ref33] Cruz-León S.; Grotz K. K.; Schwierz N. Extended magnesium and calcium force field parameters for accurate ion–nucleic acid interactions in biomolecular simulations. J. Chem. Phys. 2021, 154, 171102 10.1063/5.0048113. 34241062

[ref34] Grotz K. K.; Cruz-León S.; Schwierz N. Optimized Magnesium Force Field Parameters for Biomolecular Simulations with Accurate Solvation, Ion-Binding, and Water-Exchange Properties. J. Chem. Theory Comput. 2021, 17, 2530–2540. 10.1021/acs.jctc.0c01281. 33720710 PMC8047801

[ref35] Grotz K. K.; Schwierz N. Optimized Magnesium Force Field Parameters for Biomolecular Simulations with Accurate Solvation, Ion-Binding, and Water-Exchange Properties in SPC/E, TIP3P-fb, TIP4P/2005, TIP4P-Ew, and TIP4P-D. J. Chem. Theory Comput. 2022, 18, 526–537. 10.1021/acs.jctc.1c00791. 34881568 PMC8757469

[ref36] Grotz K. K.; Schwierz N. Magnesium force fields for OPC water with accurate solvation, ion-binding, and water-exchange properties: Successful transfer from SPC/E. J. Chem. Phys. 2022, 156, 114501 10.1063/5.0087292. 35317575

[ref37] Szabó Z. Multinuclear NMR studies of the interaction of metal ions with adenine-nucleotides. Coord. Chem. Rev. 2008, 252, 2362–2380. 10.1016/j.ccr.2008.03.002.

[ref38] Schwierz N. Kinetic pathways of water exchange in the first hydration shell of magnesium. J. Chem. Phys. 2020, 152, 224106 10.1063/1.5144258. 32534547

[ref39] Neumann J.; Schwierz N. Artificial intelligence resolves kinetic pathways of magnesium binding to RNA. J. Chem. Theory Comput. 2022, 18, 1202–1212. 10.1021/acs.jctc.1c00752. 35084846 PMC8830046

[ref40] Cunha R. A.; Bussi G. Unraveling Mg^2+^–RNA binding with atomistic molecular dynamics. RNA 2017, 23, 628–638. 10.1261/rna.060079.116. 28148825 PMC5393174

[ref41] Bai Y.; Greenfeld M.; Travers K. J.; Chu V. B.; Lipfert J.; Doniach S.; Herschlag D. Quantitative and Comprehensive Decomposition of the Ion Atmosphere around Nucleic Acids. J. Am. Chem. Soc. 2007, 129, 14981–14988. 10.1021/ja075020g. 17990882 PMC3167487

[ref42] Giambaşu G.; Luchko T.; Herschlag D.; York D.; Case D. Ion Counting from Explicit-Solvent Simulations and 3D-RISM. Biophys. J. 2014, 106, 883–894. 10.1016/j.bpj.2014.01.021. 24559991 PMC3944826

[ref43] Savelyev A.; MacKerell A. D. Competition among Li^+^, Na^+^, K^+^, and Rb^+^ Monovalent Ions for DNA in Molecular Dynamics Simulations Using the Additive CHARMM36 and Drude Polarizable Force Fields. J. Phys. Chem. B 2015, 119, 4428–4440. 10.1021/acs.jpcb.5b00683. 25751286 PMC4378841

[ref44] Yoo J.; Aksimentiev A. Competitive Binding of Cations to Duplex DNA Revealed through Molecular Dynamics Simulations. J. Phys. Chem. B 2012, 116, 12946–12954. 10.1021/jp306598y. 23016894

[ref45] Auffinger P.; Westhof E. Water and ion binding around RNA and DNA (C,G) oligomers11Edited by I. Tinoco. J. Mol. Biol. 2000, 300, 1113–1131. 10.1006/jmbi.2000.3894. 10903858

[ref46] Pasi M.; Maddocks J. H.; Lavery R. Analyzing ion distributions around DNA: sequence-dependence of potassium ion distributions from microsecond molecular dynamics. Nucleic Acids Res. 2015, 43, 2412–2423. 10.1093/nar/gkv080. 25662221 PMC4344516

[ref47] Pan F.; Roland C.; Sagui C. Ion distributions around left- and right-handed DNA and RNA duplexes: a comparative study. Nucleic Acids Res. 2014, 42, 13981–13996. 10.1093/nar/gku1107. 25428372 PMC4267617

[ref48] Gebala M.; Giambasu G. M.; Lipfert J.; Bisaria N.; Bonilla S.; Li G.; York D. M.; Herschlag D. Cation–Anion Interactions within the Nucleic Acid Ion Atmosphere Revealed by Ion Counting. J. Am. Chem. Soc. 2015, 137, 14705–14715. 10.1021/jacs.5b08395. 26517731 PMC4739826

[ref49] Gebala M.; Bonilla S.; Bisaria N.; Herschlag D. Does Cation Size Affect Occupancy and Electrostatic Screening of the Nucleic Acid Ion Atmosphere?. J. Am. Chem. Soc. 2016, 138, 10925–10934. 10.1021/jacs.6b04289. 27479701 PMC5010015

[ref50] Lavery R.; Maddocks J. H.; Pasi M.; Zakrzewska K. Analyzing ion distributions around DNA. Nucleic Acids Res. 2014, 42, 8138–8149. 10.1093/nar/gku504. 24906882 PMC4081102

[ref51] Wang J.; Xiao Y. Types and concentrations of metal ions affect local structure and dynamics of RNA. Phys. Rev. E 2016, 94, 040401 10.1103/PhysRevE.94.040401. 27841650

[ref52] Xiao S.; Liang H.; Wales D. J. The Contribution of Backbone Electrostatic Repulsion to DNA Mechanical Properties is Length-Scale-Dependent. J. Phys. Chem. Lett. 2019, 10, 4829–4835. 10.1021/acs.jpclett.9b01960. 31380654

[ref53] Kriegel F.; Matek C.; Dršata T.; Kulenkampff K.; Tschirpke S.; Zacharias M.; Lankaš F.; Lipfert J. The temperature dependence of the helical twist of DNA. Nucleic Acids Res. 2018, 46, 7998–8009. 10.1093/nar/gky599. 30053087 PMC6125625

[ref54] Long M. P.; Alland S.; Martin M. E.; Isborn C. M. Molecular dynamics simulations of alkaline earth metal ions binding to DNA reveal ion size and hydration effects. Phys. Chem. Chem. Phys. 2020, 22, 5584–5596. 10.1039/C9CP06844A. 32107511

[ref55] Robbins T. J.; Ziebarth J. D.; Wang Y. Comparison of monovalent and divalent ion distributions around a DNA duplex with molecular dynamics simulation and a Poisson-Boltzmann approach. Biopolymers 2014, 101, 834–848. 10.1002/bip.22461. 24443090 PMC4102171

[ref56] Mukherjee S.; Bhattacharyya D. Influence of divalent magnesium ion on DNA: molecular dynamics simulation studies. J. Biomol. Struct. Dyn. 2013, 31, 896–912. 10.1080/07391102.2012.713780. 22963740

[ref57] Macke T. J.; Case D. A. Modeling Unusual Nucleic Acid Structures. Molecular Modeling of Nucleic Acids 1997, 682, 379–393. 10.1021/bk-1998-0682.ch024.

[ref58] Jorgensen W. L.; Chandrasekhar J.; Madura J. D.; Impey R. W.; Klein M. L. Comparison of simple potential functions for simulating liquid water. J. Chem. Phys. 1983, 79, 926–935. 10.1063/1.445869.

[ref59] Parrinello M.; Rahman A. Polymorphic transitions in single crystals: A new molecular dynamics method. J. Appl. Phys. 1981, 52, 7182–7190. 10.1063/1.328693.

[ref60] Bussi G.; Donadio D.; Parrinello M. Canonical sampling through velocity rescaling. J. Chem. Phys. 2007, 126, 014101 10.1063/1.2408420. 17212484

[ref61] Cornell W. D.; Cieplak P.; Bayly C. I.; Gould I. R.; Merz K. M.; Ferguson D. M.; Spellmeyer D. C.; Fox T.; Caldwell J. W.; Kollman P. A. A Second Generation Force Field for the Simulation of Proteins, Nucleic Acids, and Organic Molecules. J. Am. Chem. Soc. 1995, 117, 5179–5197. 10.1021/ja00124a002.

[ref62] Pérez A.; Marchán I.; Svozil D.; Sponer J.; Cheatham T. E.; Laughton C. A.; Orozco M. Refinement of the AMBER Force Field for Nucleic Acids: Improving the Description of *α/γ* Conformers. Biophys. J. 2007, 92, 3817–3829. 10.1529/biophysj.106.097782. 17351000 PMC1868997

[ref63] Zgarbová M.; Otyepka M.; Šponer J.; Mládek A.; Banáš P.; Cheatham T. E.; Jurečka P. Refinement of the Cornell et al. Nucleic Acids Force Field Based on Reference Quantum Chemical Calculations of Glycosidic Torsion Profiles. J. Chem. Theory Comput. 2011, 7, 2886–2902. 10.1021/ct200162x. 21921995 PMC3171997

[ref64] Ivani I.; Dans P. D.; Noy A.; Pérez A.; Faustino I.; Hospital A.; Walther J.; Andrio P.; Goñi R.; Balaceanu A.; et al. Parmbsc1: a refined force field for DNA simulations. Nat. Methods 2016, 13, 55 10.1038/nmeth.3658. 26569599 PMC4700514

[ref65] Schwierz N.; Horinek D.; Netz R. R. Reversed anionic Hofmeister series: the interplay of surface charge and surface polarity. Langmuir 2010, 26, 7370–7379. 10.1021/la904397v. 20361734

[ref66] Schwierz N.; Horinek D.; Netz R. R. Anionic and cationic Hofmeister effects on hydrophobic and hydrophilic surfaces. Langmuir 2013, 29, 2602–2614. 10.1021/la303924e. 23339330

[ref67] Klein D. J.; Moore P. B.; Steitz T. A. The contribution of metal ions to the structural stability of the large ribosomal subunit. RNA 2004, 10, 1366–1379. 10.1261/rna.7390804. 15317974 PMC1370624

[ref68] Kowalczyk S. W.; Wells D. B.; Aksimentiev A.; Dekker C. Slowing down DNA Translocation through a Nanopore in Lithium Chloride. Nano Lett. 2012, 12, 1038–1044. 10.1021/nl204273h. 22229707 PMC3349906

[ref69] Kirmizialtin S.; Pabit S. A.; Meisburger S. P.; Pollack L.; Elber R. RNA and its ionic cloud: solution scattering experiments and atomically detailed simulations. Biophys. J. 2012, 102, 819–828. 10.1016/j.bpj.2012.01.013. 22385853 PMC3283807

[ref70] Pabit S. A.; Meisburger S. P.; Li L.; Blose J. M.; Jones C. D.; Pollack L. Counting ions around DNA with anomalous small-angle X-ray scattering. J. Am. Chem. Soc. 2010, 132, 16334–16336. 10.1021/ja107259y. 21047071 PMC3012602

[ref71] Rohs R.; West S. M.; Sosinsky A.; Liu P.; Mann R. S.; Honig B. The role of DNA shape in protein–DNA recognition. Nature 2009, 461, 1248–1253. 10.1038/nature08473. 19865164 PMC2793086

[ref72] Hud N. V.; Vilfan I. D. Toroidal DNA condensates: unraveling the fine structure and the role of nucleation in determining size. Annu. Rev. Biophys. Biomol. Struct. 2005, 34, 295–318. 10.1146/annurev.biophys.34.040204.144500. 15869392

[ref73] Tolokh I. S.; Pabit S. A.; Katz A. M.; Chen Y.; Drozdetski A.; Baker N.; Pollack L.; Onufriev A. V. Why double-stranded RNA resists condensation. Nucleic Acids Res. 2014, 42, 10823–10831. 10.1093/nar/gku756. 25123663 PMC4176364

[ref74] Wu Y.-Y.; Zhang Z.-L.; Zhang J.-S.; Zhu X.-L.; Tan Z.-J. Multivalent ion-mediated nucleic acid helix-helix interactions: RNA versus DNA. Nucleic Acids Res. 2015, 43, 6156–6165. 10.1093/nar/gkv570. 26019178 PMC4499160

