# Supplementary material for: RNA Captures More Cations than DNA: Insights from Molecular Dynamics Simulations
Source: J Phys Chem B. 2022 Oct 19;126(43):8646–54. doi: 10.1021/acs.jpcb.2c04488 (PMC9639116; doi:10.1021/acs.jpcb.2c04488)
Supplement: Supplementary file 1 — jp2c04488_si_001.pdf [file jp2c04488_si_001.pdf]

Supporting Information: RNA Captures more Cations than DNA: Insights from Molecular Dynamics Simulations

Sergio Cruz-León† and Nadine Schwierz*,‡,¶

†*Department of Theoretical Biophysics, Max Planck Institute of Biophysics, Max-von-Laue-Str. 3 60438 Frankfurt am Main, Germany*

‡*Institute of Physics, University of Augsburg, Universitätsstraße 1, 86159 Augsburg, Germany*

¶*Department of Theoretical Biophysics, Max Planck Institute of Biophysics, Max-von-Laue-Str. 3 60438 Frankfurt am Main, Germany*

E-mail: nadine.schwierz@physik.uni-augsburg.de

Simulation Details

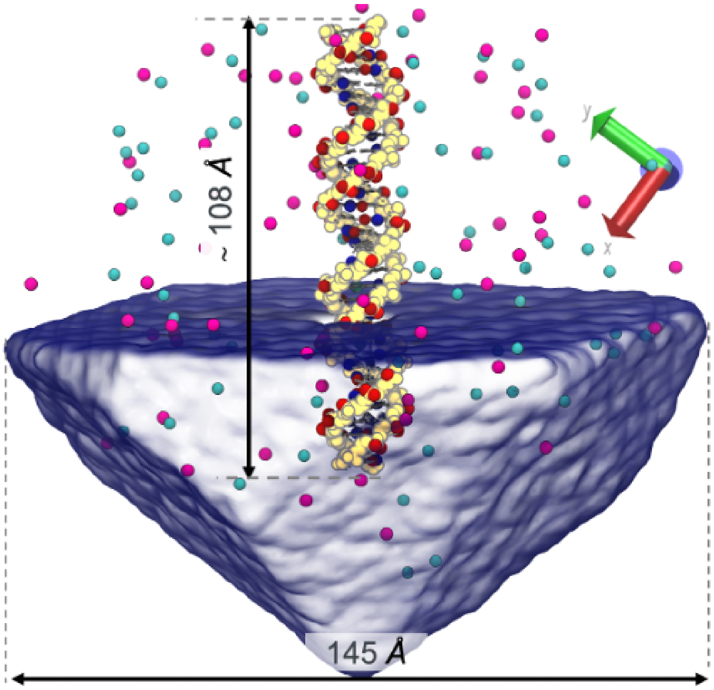

Figure S1: Typical simulation system analyzed. Data from Ref.[S1]

For all the simulations, we used the package Gromacs v.2018.1[S2] with periodic boundary conditions, and the electrostatics was treated using particle-mesh Ewald summation. The long-range electrostatic interactions were treated with cubic interpolation and a Fourier space grid of 0.12 nm. Lennard-Jones interactions and close Coulomb real space interactions were cut-off at 1.2 nm. Errors from the truncation of LJ interactions were accounted for by long-range dispersion correction for energy and pressure. The LINCS algorithm was used to constrain hydrogen bonds allowing us to use a time step of 2 fs.

Simulation protocol: Nucleic acid duplexes were placed in an orthorhombic dodecahedron box assuring a minimal distance of 2 nm to the edge and filled with TIP3P water molecules.[S3] Large boxes were required to obtain converged cylindrical concentrations. Subsequently, water molecules were randomly replaced by ions to obtain a neutral system with the desired salt bulk concentration. Afterward, a pre-equilibration protocol, consisting of energy minimization, NVT equilibration and NPT equilibration, was completed prior to

production runs. During the pre-equilibration process, the heavy atoms of the nucleic acids (NAs) were restrained (1000 kJ/(mol· nm^2)) to allow the solvent equilibration. Energy minimization used the steepest descent algorithm with a maximum of 50000 steps. Later, we employed 1 ns NVT and 1 ns NPT simulations to further equilibrate the system. NPT simulations used the isotropic Parrinello Rahman barostat[S4] with a coupling constant of 5.0 ps. The temperature was maintained using the velocity rescaling thermostat with a stochastic term[S5] and a coupling constant of 0.1 ps. Finally, production runs were performed in the NPT ensemble with 3 μs long trajectories for monovalent cations and 5 μs long trajectories for divalent cations. For the analysis, the first 200 ns were discarded for equilibration.

Two-dimensional untwisted distributions projected on the x-y plane

The plots in Figure 3B,D and Figure 4B,D were obtained from the software `canion`.[S6] The MD trajectories were first preprocessed with `Curves+`.[S7] Afterward, we read the radial concentration as a function of the angle output generated by canion (.ra file) and perform a coordinate transformation to project them on Cartesian coordinates. For better visualization, we resampled it in a 0.15 Å grid. The resampling was done with the matplotlib.tri triangulation package of python. A jupyter notebook and a minimal example to reproduce Figure 3B is freely available in the following repository: `https://github.com/sergiocruzleon/Plotting_Canion_output`

Concentration Profiles

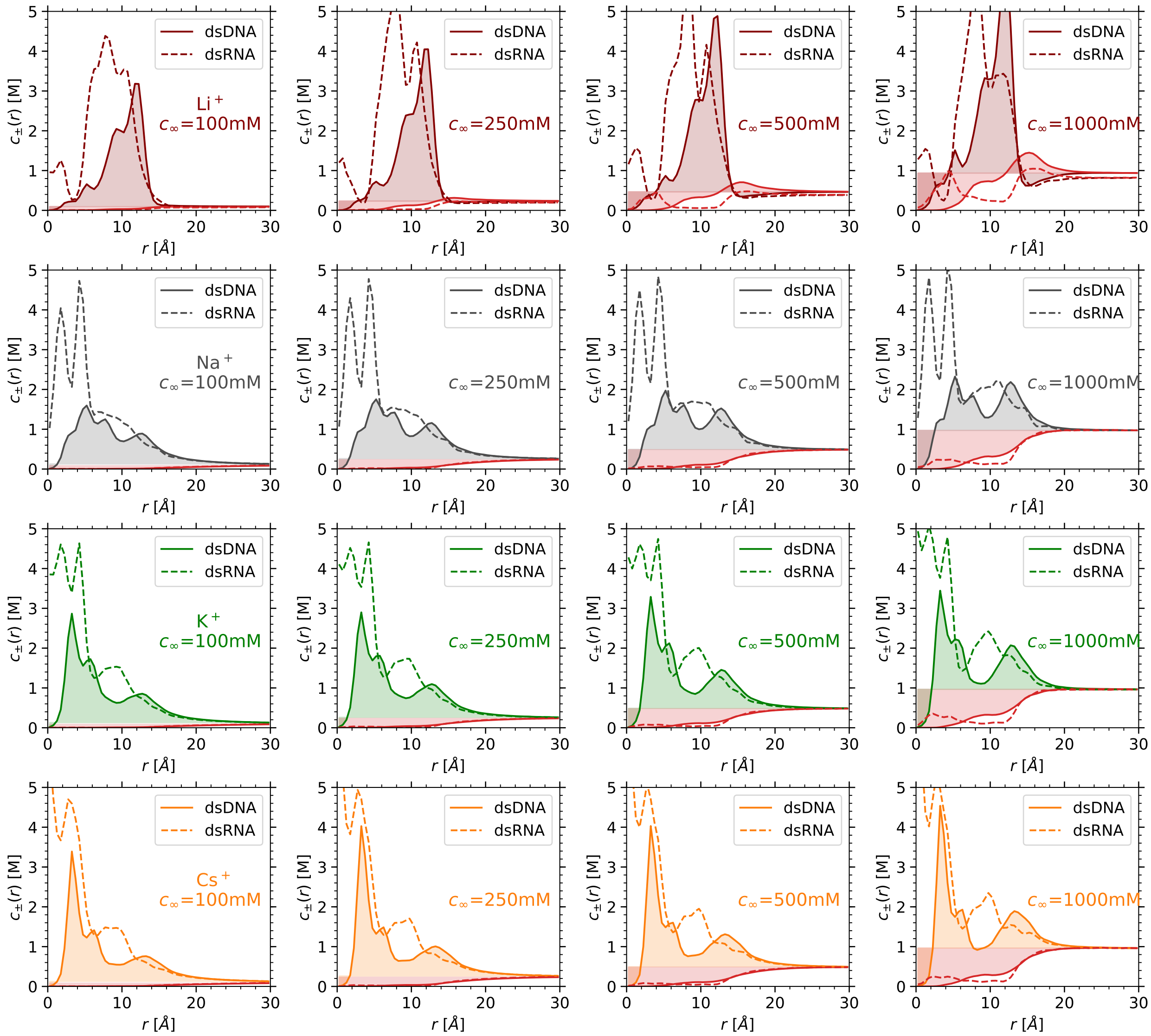

Figure S2: Ion concentration profiles $c_{\pm}$ for monovalent cations as function of distance r for different bulk salt concentrations: 100 mM, 250 mM, 500 mM, 1000 mM. Solid lines correspond to the results for DNA, dahsed lines correspond to the results for RNA. The red solid lines shows the concentration profile of the co-ion Cl^{-}.

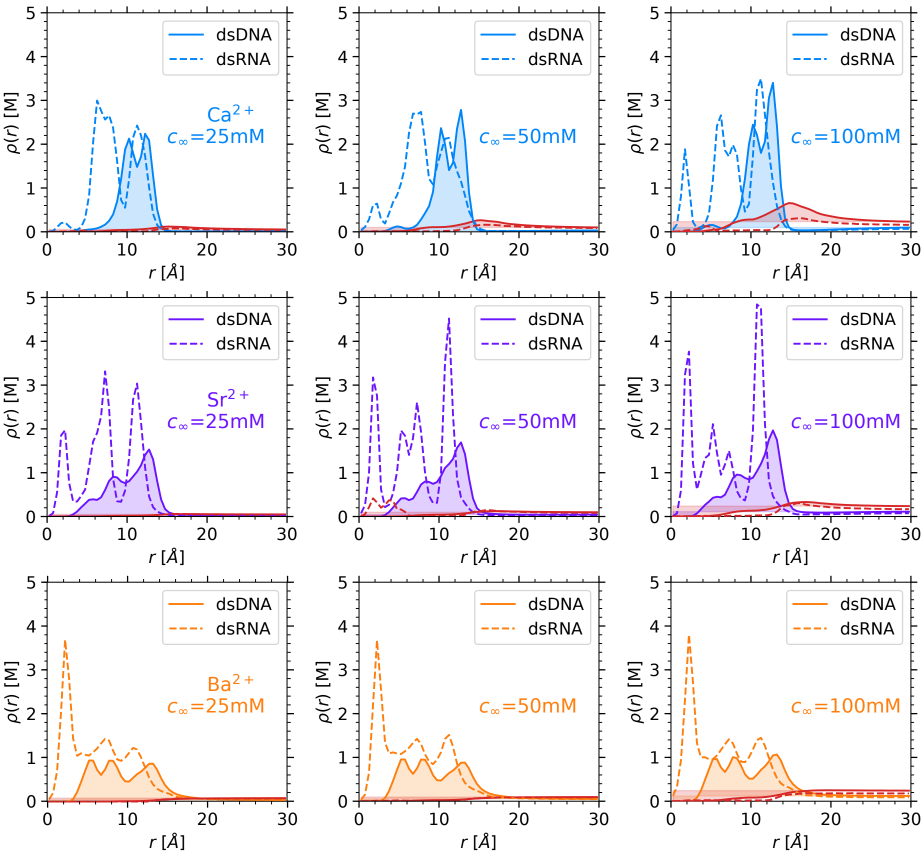

Figure S3: Ion concentration profiles $c_{\pm}$ for divalent cations as function of distance r for different bulk salt concentrations: 25 mM, 50 mM, 100 mM. Solid lines correspond to the results for DNA, dahsed lines correspond to the results for RNA. The red solid lines shows the concentration profile of the co-ion Cl^-.

Cutoff distance to define inner-sphere binding

Table S1: Cutoff distance $d^\dagger$ to define inner-sphere binding of the different cations at the various binding sites. The values are based on the free energy profiles obtained from umbrella sampling in our previous work.[S8]

Cation	O1P and O2P $d^\dagger$ [nm]	N7 $d^\dagger$ [nm]	O2, O4, O6 O3′ O4′ and O5′ $d^\dagger$ [nm]
Li^+	0.25	0.28	0.25
Na^+	0.30	0.35	0.30
K^+	0.35	0.36	0.34
Cs^+	0.40	0.40	0.37
Ca^{2+}	0.30	0.32	0.32
Sr^{2+}	0.31	0.33	0.32
Ba^{2+}	0.33	0.36	0.34

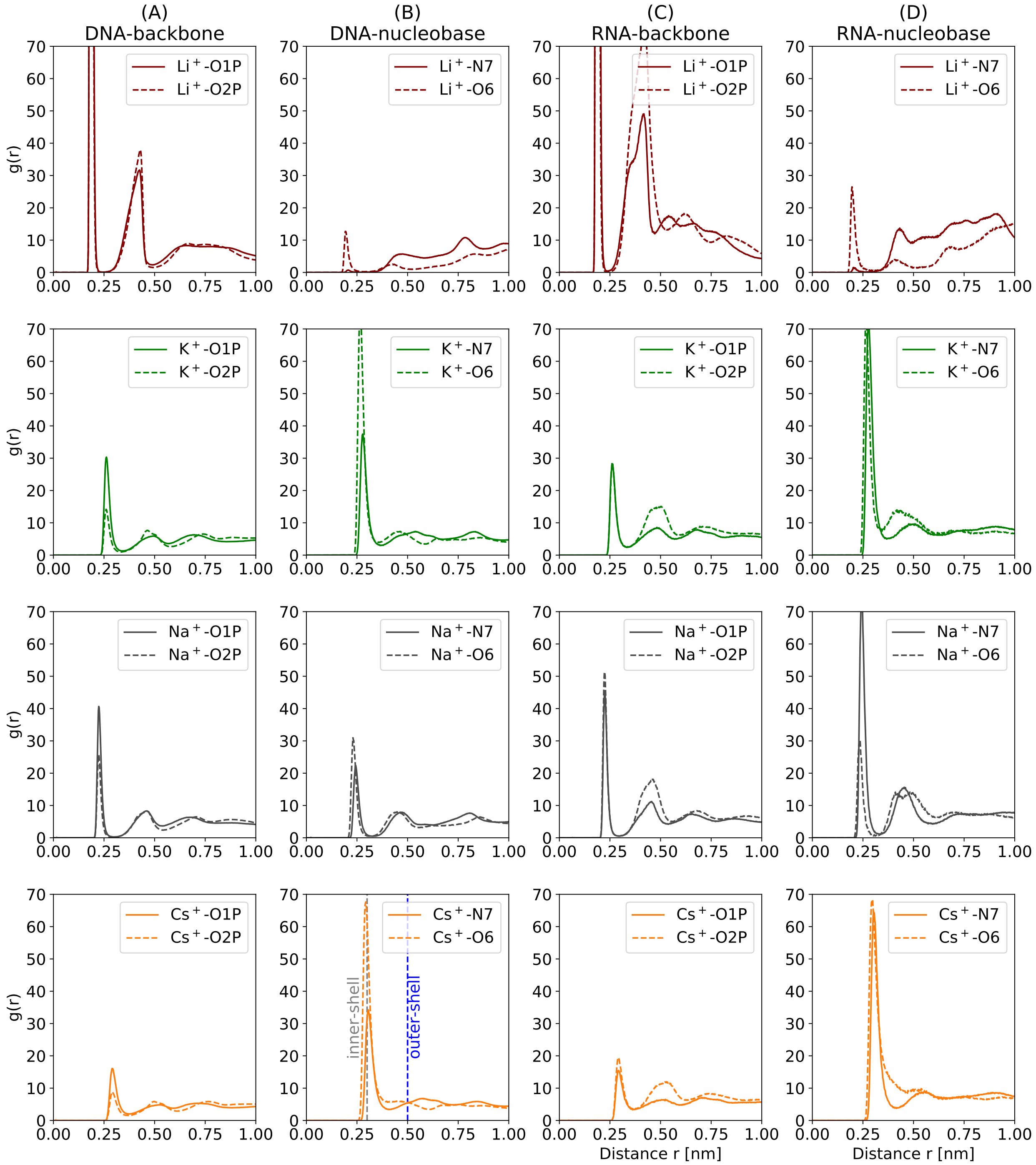

Figure S4: Radial distribution function $g(r)$ for monovalent cations as function of distance r for the main binding sites on the backbone (O1P and O2P) and at the nucleobase (N7 and O6). Results for 100 mM.

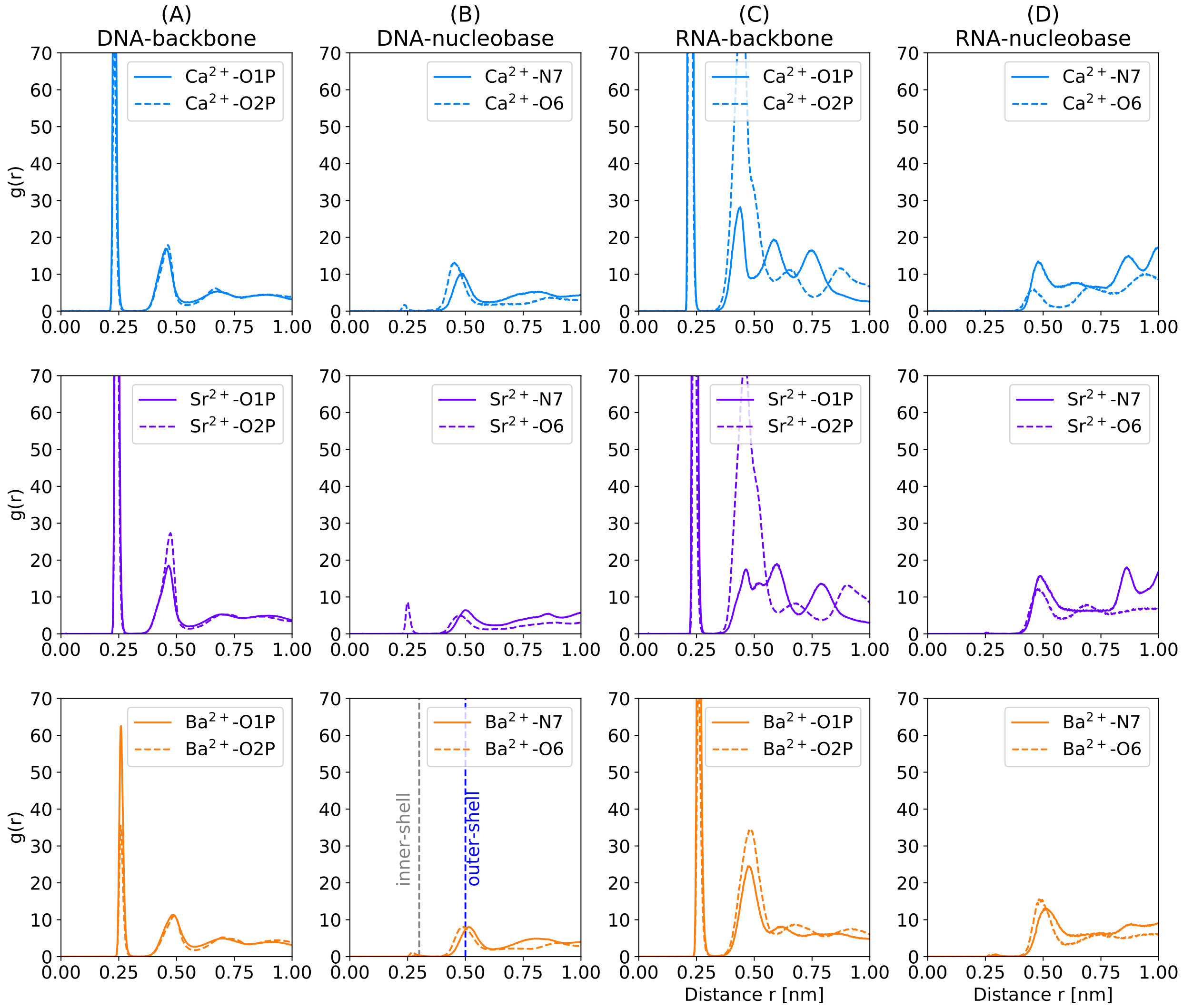

Figure S5: Radial distribution function $g(r)$ for divalent cations as function of distance r for the main binding sites on the backbone (O1P and O2P) and at the nucleobase (N7 and O6). Results for 100 mM.

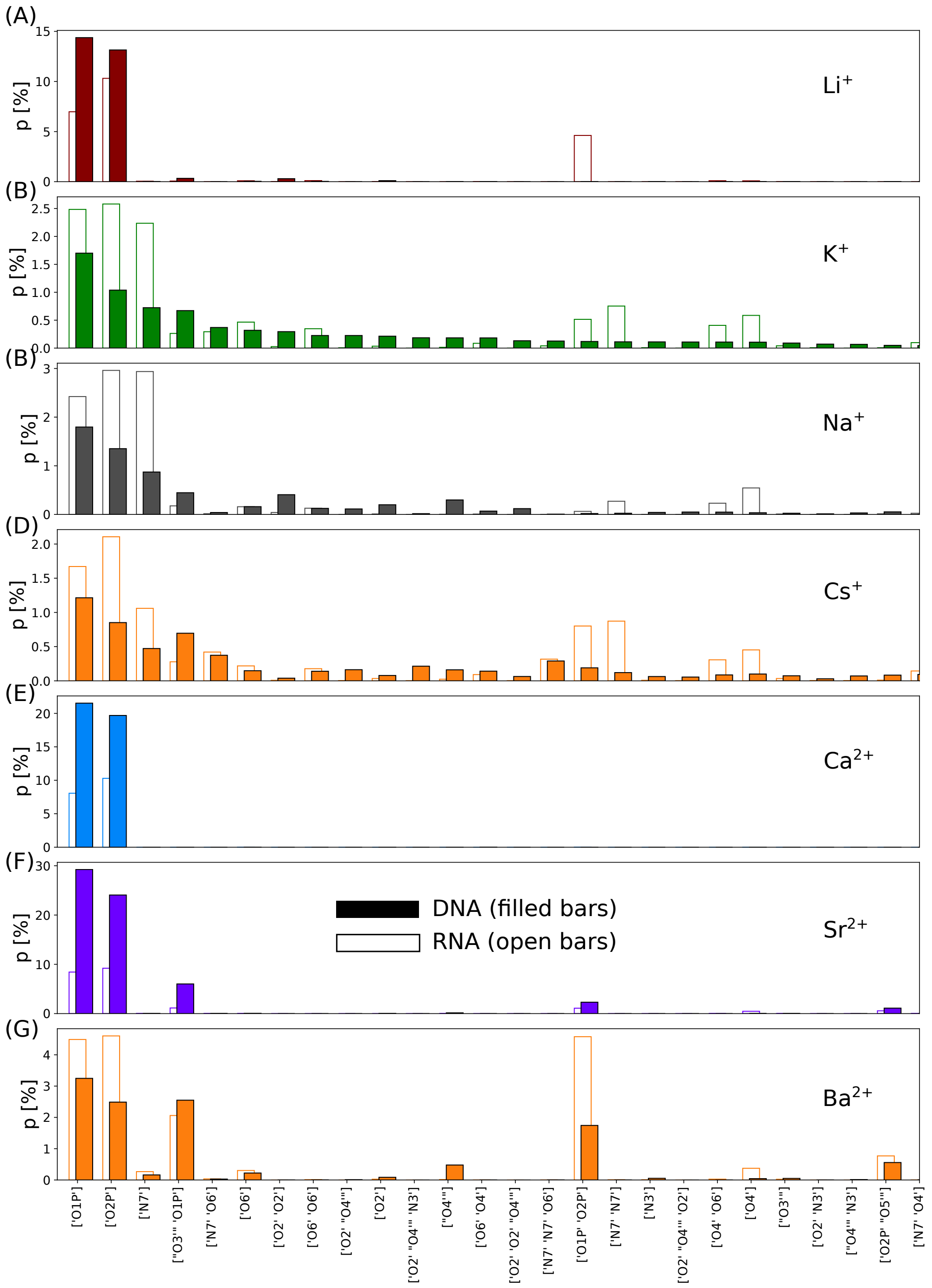

Figure S6: Probability p of a given binding pattern for DNA and RNA. For comparison among cations, the probabilities of all ions are ordered with the top 26 patterns for K^+ interacting with DNA.

References

(S1) Cruz-León, S.; Vanderlinden, W.; Müller, P.; Forster, T.; Staudt, G.; Lin, Y.-Y.; Lipfert, J.; Schwierz, N. Twisting DNA by salt. *Nucleic Acids Res.* **2022**, *50*, 5726–5738.

(S2) Abraham, M. J.; Murtola, T.; Schulz, R.; Páll, S.; Smith, J. C.; Hess, B.; Lindahl, E. GROMACS: High performance molecular simulations through multi-level parallelism from laptops to supercomputers. *SoftwareX* **2015**, *1-2*, 19 – 25.

(S3) Jorgensen, W. L.; Chandrasekhar, J.; Madura, J. D.; Impey, R. W.; Klein, M. L. Comparison of simple potential functions for simulating liquid water. *J. Chem. Phys.* **1983**, *79*, 926–935.

(S4) Parrinello, M.; Rahman, A. Polymorphic transitions in single crystals: A new molecular dynamics method. *J. Appl. Phys.* **1981**, *52*, 7182–7190.

(S5) Bussi, G.; Donadio, D.; Parrinello, M. Canonical sampling through velocity rescaling. *J. Chem. Phys.* **2007**, *126*, 014101.

(S6) Pasi, M.; Maddocks, J. H.; Lavery, R. Analyzing ion distributions around DNA: sequence-dependence of potassium ion distributions from microsecond molecular dynamics. *Nucleic Acids Res.* **2015**, *43*, 2412–2423.

(S7) Lavery, R.; Maddocks, J. H.; Pasi, M.; Zakrzewska, K. Analyzing ion distributions around DNA. *Nucleic Acids Res.* **2014**, *42*, 8138–8149.

(S8) Cruz-León, S.; Schwierz, N. Hofmeister Series for Metal-Cation–RNA Interactions: The Interplay of Binding Affinity and Exchange Kinetics. *Langmuir* **2020**, *36*, 5979–5989.
